# Estimating the prevalence of Epstein–Barr virus in primary gastric lymphoma: a systematic review and meta-analysis

**DOI:** 10.1186/s13027-023-00482-2

**Published:** 2023-02-10

**Authors:** Mayo Hirabayashi, Alexandra Traverse-Glehen, Jean-Damien Combes, Gary M. Clifford, Catherine de Martel

**Affiliations:** 1 Early Detection, Prevention and Infections Branch, International Agency for Research on Cancer (IARC/WHO), 25 avenue Tony Garnier, CS 90627, 69633 Lyon CEDEX 07, France; 2 Hospices Civils de Lyon, Institut de Pathologie Multisite, Hôpital Lyon-Sud, Pierre Bénite, France; 3 Centre International de Recherche en Infectiologie (CIRI) INSERM U1111 - CNRS UMR5308, Université Claude Bernard Lyon I - ENS de Lyon, Lyon, France

**Keywords:** Gastric lymphoma, Epstein–Barr virus, *Helicobacter pylori*

## Abstract

The stomach is a common site for extranodal non-Hodgkin’s lymphoma. While *Helicobacter pylori* (*H. pylori*) is the main established risk factor for primary gastric lymphoma, a fraction could be aetiologically associated with Epstein–Barr virus (EBV), a known haematolymphoid carcinogen. We systematically searched five databases from 1 January 1990 until 31 May 2022 for studies reporting EBV prevalence in gastric lymphoma tumour tissue by in-situ hybridisation (ISH) for EBV-encoded small RNA (PROSPERO CRD42020164473). We included representative series of more than five gastric lymphoma cases. Pooled prevalence and corresponding 95% confidence intervals (CI) of EBV in gastric tumour cells were calculated for two major gastric B-cell lymphoma types, mucosa-associated lymphoid tissue (MALT) lymphoma and diffuse large B-cell lymphoma (DLBCL). When available, we also extracted data on *H. pylori* prevalence and survival by EBV status. We found ten studies including 194 cases of gastric MALT lymphoma and 11 studies including 643 cases of gastric DLBCL. EBV prevalence was 2.2% (95% CI: 0.5–13.3) in gastric MALT lymphoma and 11.0% (95% CI: 5.2–20.0) in gastric DLBCL. In a subset of studies, the prevalence of *H. pylori* was higher in gastric MALT lymphoma (51/69) compared to gastric DLBCL (62/102). Overall, our findings suggest that EBV is rarely seen in MALT lymphoma but is associated with around 10% of gastric DLBCL, similar to the proportion observed at other primary sites. EBV-related lymphoma adds a small number of cases to the burden of cancer that could be prevented by the future development of a vaccine against EBV.

**Supplementary Information:**

The online version contains supplementary material available at 10.1186/s13027-023-00482-2.

## Introduction

The stomach is the most common primary site for extranodal non-Hodgkin lymphoma (NHL) [1] although primary gastric lymphoma constitutes less than 5% of all primary gastric neoplasms [2]. While classification of lymphoma is complex and constantly evolving, there are two major histological subtypes of primary gastric lymphoma: mucosa-associated lymphoid tissue (MALT) lymphoma and diffuse large B-cell lymphoma (DLBCL). As described in the WHO classification, MALT lymphoma is an extranodal low-grade B-cell lymphoma composed of morphologically heterogeneous small B lymphocytes, including characteristic centrocyte-like cells [3]. DLBCL is more aggressive, and characterised by the presence of compact aggregates or a sheet-like proliferation of the large cell [4]. Based on its morphology, DLBCL can be subdivided into the centroblastic subtype which is more common and has better prognosis, and the immunoblastic subtype which is considered more aggressive [5].

Infection, immunosuppression after solid organ transplantation, celiac disease and inflammatory bowel disease are established risk factors for gastric lymphoma [6]. Although limited epidemiological studies have been conducted, a causal relationship between *Helicobacter pylori* (*H. pylori*), a carcinogenic agent and gastric lymphoma is widely accepted [7]. In the 1990s, up to 90% gastric MALT lymphoma patients were reported to be infected with *H. pylori* and clinical studies [8, 9] showed complete remission in 70% of *H. pylori-*positive gastric MALT lymphoma patients when *H. pylori* was treated. Cases of *H. pylori*-negative gastric lymphoma have increased in recent years, however, especially in Western countries, suggesting other possible risk factors [10].

Epstein–Barr Virus (EBV) is a ubiquitous oncogenic virus known to infect only humans [11] and latent asymptomatic infection has been reported among 90% of the global adult population [12]. EBV is aetiologically linked to several lymphoid malignancies, including Burkitt lymphoma, Hodgkin lymphoma, DLBCL, as well as several types of T/NK-cell lymphoma and nasopharyngeal carcinoma [12]. In 2017, a publication by the Cancer Genome Atlas Project showed a distinct molecular subtype of EBV-associated gastric adenocarcinoma [13]. While several meta-analyses have described the prevalence of EBV in gastric adenocarcinoma, the association between EBV and gastric lymphoma has rarely been studied. The presence of EBV in gastric tumour cells could be an indication of a causal relationship between EBV and stomach lymphoma. We explore here the prevalence of EBV in primary gastric lymphoma as part of a global work programme to monitor and update estimates of the burden of cancer attributable to infectious diseases.

## Materials and methods

We conducted a systematic literature review and meta-analysis to determine the presence of EBV in primary gastric lymphoma tumour tissue. The study was carried out according to PRISMA reporting and has been registered in PROSPERO (registration number: CRD42020164473).

### Search strategy and study selection

We performed a systematic search on the available literature in PubMed (MEDLINE), Scopus, Web of Science, Embase, and SciELO, without language restriction, only considering original articles from 1 Jan 1990, after the introduction of in-situ hybridisation (ISH) for EBV-encoded RNA (EBER1 and 2) [14], until 31 May 2022. Duplicate records were removed. Titles and abstracts, and then full texts were screened by two researchers (MH and CdM). Reference lists of included articles were also reviewed for relevant material. Any disagreements or clarification of the inclusion criteria were settled through discussion. Search strategies are provided in Additional file 1: Data 1*.*

### Eligibility criteria

Articles were included if they were published in peer-reviewed journals, cases were unselected and representative, and the presence of EBV was assessed in tumour tissue using the ISH technique targeting EBER − 1 or − 2.

### Data extraction

The following data were abstracted when reported: first author; year of publication; journal; country; geographical region [15]; name of hospital; sample size; age; sex; gastric lymphoma morphologic subtype: MALT lymphoma (low-/high-grade), DLBCL (centroblastic/immunoblastic); survival; *H. pylori* detection method; *H. pylori* status; proportion of *H. pylori*-positive samples; and proportion of EBV-positive samples by ISH.

### Statistical analysis

We calculated pooled EBV prevalence, 95% confidence intervals (CI), 95% prediction intervals (PI), heterogeneity (I^2^), and between-study variance (τ^2^), using the random intercept logistic regression model [16]. Separate estimates were reported for gastric MALT lymphoma and DLBCL.

Exact binomial test was used to calculate prevalence and corresponding 95% CIs of *H. pylori* in gastric lymphomas for each study. Analyses were performed using R statistical software (version 4.0.4, RStudio: Integrated Development for R, Boston, MA, USA); packages Meta and Metafor.

## Results

Our systematic review identified 7,354 papers from five databases (Additional file 1: Fig. S1). After excluding studies not meeting our inclusion criteria based on titles and abstracts, we retrieved 1,072 full articles to be considered for inclusion. Twelve articles investigating the presence of EBV in gastric MALT lymphoma or gastric DLBCL using ISH were selected for the final analysis [17–28].

### Gastric MALT lymphoma

**Fig. 1:**
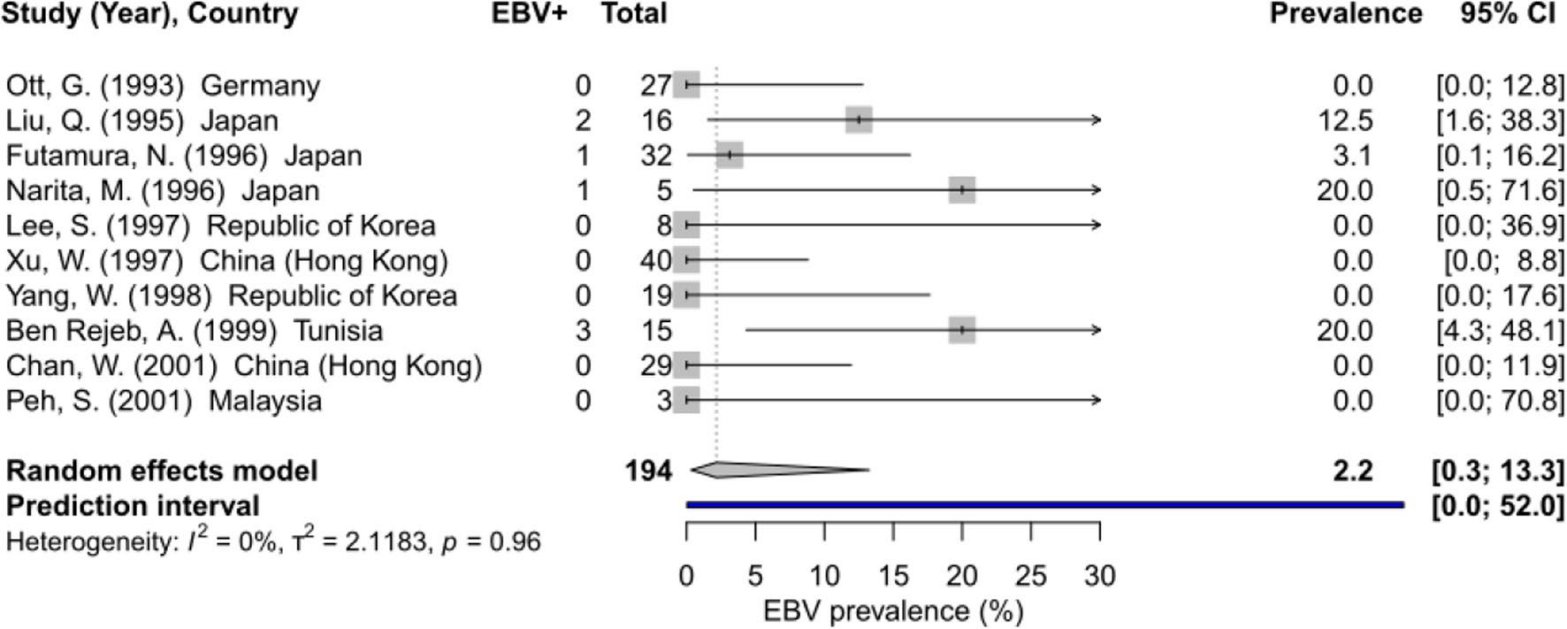
Global EBV prevalence in MALT lymphoma. EBV = Epstein–Barr virus. I^2^ = percentage of variation across studies due to heterogeneity rather than chance

Ten studies (Eastern Asia (n = 7), Europe (1), North Africa (1), South-Eastern Asia (1)) with 194 cases from six countries reported EBV prevalence by ISH in gastric MALT lymphoma (Table 1). The pooled EBV prevalence in gastric MALT lymphoma was 2.2% (95% CI 0.3–13.3, I^2^ = 0.0%) (Fig. 1). Five studies reported EBV prevalence in low- versus high-grade MALT lymphoma. EBV prevalence in 63 low-grade lymphoma cases was 1.6% (95% CI 0.1–20.9, I^2^ = 0.0%), while the prevalence of EBV in 60 high-grade cases was zero, with no statistical difference.

**Table 1:** Studies on the detection of Epstein–Barr virus by ISH in patients with primary gastric lymphoma

Reference (Year), Country	Threshold for EBV positivity by ISH (% of tumour cells)	Gastric MALT lymphoma			Gastric DLBCL		
		EBV positive/total	EBV positive/ total by subtype	*H. pylori* positive/total (by subtype)	EBV positive/total	EBV positive/ total by morphology	*H. pylori* positive/total
Ott et al. [17] (1993), Germany	N/A	0/27	N/A	N/A	2/24	Centroblastic: 2/22 Immunoblastic: 0/2	N/A
Liu et al. [18] (1995), Japan	N/A	2/16	N/A	N/A	2/33	Centroblastic:2/30 Immunoblastic:0/3	N/A
Futamura et al. [19] (1996), Japan	≥ 5	1/32	Low grade: 1/23 High grade: 0/9	N/A	1/9	N/A	N/A
Narita et al. [20] (1996), Japan	N/A	1/5	N/A	N/A	1/8	N/A	N/A
Lee et al. [21] (1997), Republic of Korea	> 50	0/8	N/A	N/A	5/43	N/A	N/A
Xu et al. [22] (1997), China (Hong Kong)	N/A	0/40	Low grade: 0/13 High grade: 0/27	22/40 Low grade:8/13 High grade: 14/27	1/13	N/A	7/13
Yang et al. [23] (1998), Republic of Korea	N/A	0/19	Low grade: 0/12 High grade: 0/7	N/A	1/13	N/A	N/A
Ben Rejeb et al. [24] (1999), Tunisia	N/A	3/15	N/A	N/A	N/A	N/A	N/A
Chan et al. [25] (2001), China (Hong Kong)	> 90	0/29	Low grade: 0/14 High grade: 0/15	29/29	10/17	N/A	6/17
Peh [26] (2001), Malaysia	> 50	0/3	Low grade: 0/1 High grade: 0/2	N/A	0/7	N/A	N/A
Ishikawa et al. [27] (2019), Japan	≥ 80	N/A	N/A	N/A	25/240	N/A	49/72
Zhou et al. [28] (2020), China	> 80	N/A	N/A	N/A	22/236	N/A	N/A

EBV = Epstein–Barr virus; *H. pylori* = *Helicobacter pylori*; MALT lymphoma = mucosa-associated lymphoid tissue lymphoma; DLBCL = diffuse large B-cell lymphoma; ISH = in-situ hybridisation; N/A = information not available

### Gastric DLBCL

**Fig. 2:**
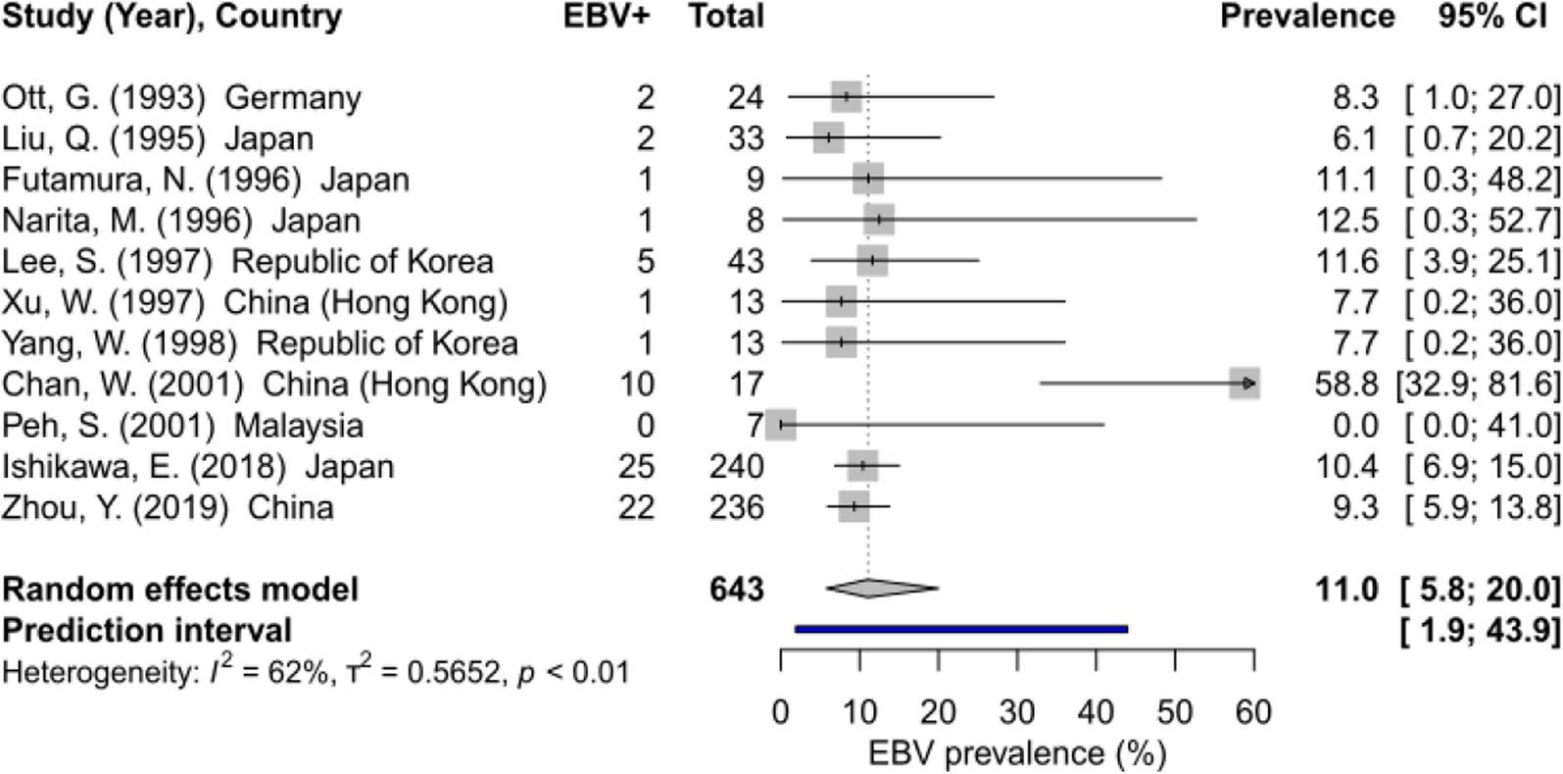
Global EBV prevalence in DLBCL. EBV = Epstein–Barr virus. I^2^ = percentage of variation across studies due to heterogeneity rather than chance

Eleven studies (Eastern Asia (n = 9), Europe (1), South-Eastern Asia (1)) with 643 cases from five countries reported EBV prevalence in gastric DLBCL (Table 1). The pooled EBV prevalence was 11.0% (95% CI 5.8–20.0, I^2^ = 62%) (Fig. 2). EBV prevalence in gastric DLBCL by morphologic subtypes was described in two studies reporting on 57 cases (Table 1). EBV prevalence in centroblastic DLBCL (two studies, 52 cases) was 8% (95% CI: 0.0–98.4), while among five immunoblastic DLBCL cases, it was zero, with no statistical difference between the two subtypes.

### H. pylori prevalence in gastric lymphoma

Two studies on gastric MALT lymphoma and three studies on gastric DLBCL reported on both EBV and *H. pylori* prevalence (Table 1). In total, *H. pylori* was found in 51 out of 69 gastric MALT lymphoma cases: 55.0% and 100% of the two studies, respectively. *H. pylori* was found in 62 out of 102 DLBCL cases: 54%, 35% and 68% of the three studies, respectively (Table 1).

We studied *H. pylori* prevalence in gastric lymphoma by EBV status. In patients with gastric MALT lymphoma, none of the patients tested for *H. pylori* were reported EBV-positive. Two studies reported on *H. pylori* prevalence in gastric DLBCL by EBV status and did not find a statistically different proportion between the two groups. The first study tested 7 out of 25 EBV-positive DLBCL cases and 65 out of 215 EBV-negative DLBCL cases for *H. pylori*. The prevalence was 71.0% (95% CI: 29.0–96.3) in EBV-positive DLBCL (5 out of 7) and 67.7% (95% CI: 54.9–78.8) in EBV-negative DLBCL (44 out of 65). In the second smaller study, the prevalence was 20.0% (95% CI: 2.5–55.6) in EBV-positive (2 out of 10) versus 57.1% (95% CI: 18.4–90.1) in EBV-negative DLBCL (4 out of 7).

### Survival

Two eastern Asian studies on gastric DLBCL reported no statistical difference in overall survival by EBV status.

## Discussion

We present the results of a systematic review and meta-analysis estimating the pooled EBV prevalence in gastric lymphoma patients based on studies published in the past 30 years. The prevalence of EBV in 10 gastric MALT lymphoma and 11 DLBCL studies was 2.2% and 11%, respectively. Our results suggest that EBV does not play a significant role in MALT lymphopathogenesis, while it might be causally associated with a small proportion of DLBCL. In a small subset of studies, *H. pylori* prevalence appeared to be higher in gastric MALT lymphoma compared to DLBCL, with no demonstrated statistical difference by EBV status.

The overall prevalence of EBV found in gastric DLBCL (11%; 95% CI 5.8–20.0) is similar to that reported in a recent meta-analysis of 31 DLBCL studies. The authors observed a pooled prevalence of 7.9% (95% CI: 5.8–9.6), irrespective of the primary site [29]. Of note, this proportion is also similar to that described in gastric adenocarcinoma in a recent meta-analysis by our group (7.5%; 95% CI: 6.9–8.1) [30]. While gastric adenocarcinoma and gastric DLBCL are different diseases, they both add to the burden of potentially preventable EBV-related cancers.

Our results, mostly based on two recent large Asian studies, suggest that the stomach is no different from other primary sites for EBV-associated lymphoma. Although we expected to see more EBV-associated tumours in immunoblastic than in centroblastic DLBCL, as in immunocompromised patients [31], the results from two studies regrouping 57 DLBCL patients, but only 5 of the immunoblastic histological subtype, showed no statistical difference between subtypes. This may be due to the characteristics of the population studied (mostly immunocompetent adults) and/or to the small sample size.

In this review, EBV was seldom detected in gastric MALT lymphoma tumour tissue. Some studies distinguished between low- and high-grade lymphoma, yet our sub-analyses did not find a difference between the two groups, both being generally negative for EBV. Arguments against this grading have been raised over the years and there is no consensus on the definition of high-grade MALT lymphoma. It is sometimes defined as compact clusters of sheets of large atypical lymphoid cells with DLBCL-like cells observed in at least 10% or more of the neoplastic lymphoid population [32]. In 1997, WHO suggested that high-grade MALT lymphoma should be classified as DLBCL [33]. Such a reclassification would have yielded a slightly lower prevalence of EBV in DLBCL patients in our analysis. Furthermore, in the subset of studies presenting data on low- versus high-grade MALT lymphoma, prevalence was lower (< 1%) than in the whole group (2.2%). By adding an extra step of histological grading, this subset of studies may have been of higher quality. This would suggest that despite the main statistical analysis showing no heterogeneity among all studies, the presence of smaller, lesser quality studies with higher prevalence could have led to a slightly overestimated prevalence of EBV in MALT lymphoma patients, and that the true prevalence might be even lower than 2.2%.

A limited number of epidemiological studies have been conducted on the association between *H. pylori* infection and gastric lymphoma. In patients with gastric MALT lymphoma, the first large study in which this association was examined histologically found the organism in over 90% of cases [34]. Subsequent studies have generally shown a lower incidence consistent with our findings (51 out of 69 cases) although these should be interpreted carefully as they are based on only two studies that examined the presence of both EBV and *H. pylori*.

In this study, we observed lower frequency of *H. pylori* in gastric DLBCL (62 out of 102 cases) compared to gastric MALT lymphoma (51 out of 69). We did not provide point estimates, nor did we test for statistical significance as data came from the very small number of studies that tested for both infectious agents (EBV and *H. pylori*) histologically, at the same time. These studies are probably not representative of all studies that have looked at the presence of *H. pylori* in patients with gastric MALT or DLBC lymphoma. The order of magnitude, however, is consistent with that found in the literature. Based on very few studies, we found no evidence that the prevalence of *H. pylori* in gastric lymphoma varies by EBV status, but the lack of data and the heterogeneity of the results in the available studies prevent us from drawing any decisive conclusion on this issue. While there is some evidence of combined involvement of EBV and *H. pylori* in the development of gastric carcinoma [35], this extensive review could not identify any study exploring a potential interaction of the two infectious agents in gastric lymphoma. This field would certainly warrant further research.

Our study has several strengths. To our knowledge, this is the only meta-analysis and systematic review conducted on the association of EBV with gastric MALT lymphoma and DLBCL. We searched five databases, applied no language restriction and only included studies that detected EBER in tumour cells by ISH, the gold standard for detecting and localising EBV in tumour tissue [36].

There has clearly been little research in this field, especially over recent years, and the resulting lack of data has led to several limitations of our study. All studies but two came from Asia meaning that we could not explore possible variations by geographical region. This unbalanced geographical distribution probably reflects a greater interest of researchers in Asia in this topic due to the higher incidence of gastric cancer and other EBV-related cancer such as nasopharyngeal carcinoma in Asia [37]. An attempt to retrieve age and sex data was made. Unfortunately, lack of data on sex and inconsistent age grouping prevented us from undertaking stratification analyses using these variables. In several analyses, small sample sizes led to wide ranges of 95% CIs for EBV prevalence and inconclusive results, despite different point estimates. Another limitation is linked to the fact that there is no established EBER cut-off for gastric lymphoma and that different thresholds were used to define EBV positivity in different studies. This may have influenced EBV-positive proportions, especially in DLBCL. A standardised threshold might allow the estimation of EBV prevalence with increased accuracy and better comparison among studies [38]. Lastly, data on lymphoma survival were too limited and heterogeneous to allow their analysis and/or interpretation.

In conclusion, while the burden of gastric lymphoma attributable to EBV seems to be small and to only concern the DLBCL histological subtype, more studies are certainly needed to better explore a possible role for the virus in lymphomagenesis, either alone or in interaction with *H. pylori*. There is great potential for reducing the burden of EBV-associated diseases through primary prevention, from infectious mononucleosis infection to cancer, and an increased interest in the development of EBV vaccines has been seen in the past decade. Such a vaccine may hold the key to preventing the entire spectrum of EBV-associated neoplasms, including some EBV-associated gastric DLBCL.

## Supplementary Information

**Additional file 1. Data 1**: Search Strategy. **Figure S1**: Inclusion and exclusion criteria flow chart.

## Data Availability

The dataset supporting the conclusions of this article will be made available upon reasonable request to the corresponding author.

## Abbreviations

CI: Confidence interval
DLBCL: Diffuse large B-cell lymphoma
EBER ISH: EBV-encoded RNA in-situ hybridization
EBV: Epstein–Barr virus
*H. pylori*: *Helicobacter pylori*
MALT: Mucosa-associated lymphoid tissue lymphoma
PI: Prediction interval
PR: Prevalence ratio
