# Supplementary material for: Estimating the prevalence of Epstein–Barr virus in primary gastric lymphoma: a systematic review and meta-analysis
Source: Infect Agent Cancer. 2023 Feb 10;18:8. doi: 10.1186/s13027-023-00482-2 (PMC9912516; doi:10.1186/s13027-023-00482-2)
Supplement: Supplementary file 1 — Additional file 1. Data 1: Search Strategy. Figure S1: Inclusion and exclusion criteria flow chart. [file 13027_2023_482_MOESM1_ESM.docx]

**ADDITIONAL FILE 1**

[ADDITIONAL Data 1: Search Strategy 2](#_Toc110418356)

[Embase 2](#_Toc110418357)

[PubMed 3](#_Toc110418358)

[Scopus 3](#_Toc110418359)

[SciELO 3](#_Toc110418360)

[World of Science 4](#_Toc110418361)

[ADDITIONAL Figure 1: Inclusion and exclusion criteria flow chart 5](#_Toc110418362)

Additional Data 1: Search Strategy

Embase

| **Search**  **Number** | **Search terms** |
| --- | --- |
| #14 | #12 OR #13 |
| #13 | ('epstein barr virus associated stomach lymphoma':ti,ab,kw OR 'epstein barr virus associated gastric lymphoma ':ti,ab,kw) |
| #12 | #5 AND #11 |
| #11 | #9 OR #10 |
| #10 | epstein barr virus'/de |
| #9 | #6 OR #7 OR #8 |
| #8 | ('infectious mononucleosis':ti,ab,kw OR 'infectious mononucleosis virus*':ti,ab,kw) |
| #7 | ('human herpesvirus 4*':ti,ab,kw OR 'human herpes virus 4*':ti,ab,kw OR 'hhv 4*':ti,ab,kw) |
| #6 | ('epstein barr virus*':ti,ab,kw OR 'ebv*':ti,ab,kw OR 'epv*':ti,ab,kw OR 'eb virus*':ti,ab,kw OR 'epstein virus*':ti,ab,kw) |
| #5 | #1 OR #2 OR #3 OR #4 |
| #4 | ('stomach tumor'/de OR 'stomach lymphoma'/de) |
| #3 | ('gastric mucosa-associated lymphoid tissue lymphoma':ti,ab,kw OR 'stomach mucosa-associated lymphoid tissue lymphoma':ti,ab,kw OR 'gastric MALT':ti,ab,kw OR 'stomach MALT':ti,ab,kw) |
| #2 | ('gastric Diffused large B-cell lymphoma':ti,ab,kw OR 'stomach Diffused large B-cell lymphoma':ti,ab,kw OR 'gastric DLBCL':ti,ab,kw OR 'stomach DLBCL':ti,ab,kw) |
| #1 | ('gastric lymphoma':ti,ab,kw OR 'stomach lymphoma':ti,ab,kw) |

PubMed

((((epstein barr virus associated gastric lymphoma[Title/Abstract]) OR (epstein barr virus associated stomach lymphoma[Title/Abstract])) OR (epstein barr virus associated gastric lymphoma[Text Word])) OR (epstein barr virus associated stomach lymphoma[Text Word])) OR (((((((((((((((epstein barr virus[Title/Abstract]) OR (human herpesvirus 4[Title/Abstract])) OR (infectious mononucleosis[Title/Abstract])) OR (human herpes virus 4[Title/Abstract])) OR (ebv[Title/Abstract])) OR (eb virus[Title/Abstract])) OR (epstein barr virus[Text Word])) OR (human herpesvirus 4[Text Word])) OR (human herpes virus 4[Text Word])) OR (ebv[Text Word])) OR (eb virus[Text Word])) OR (infectious mononucleosis[Text Word])) OR (herpesvirus 4 infections, human[MeSH Terms])) OR (herpesvirus 4, human[MeSH Terms])) AND (((((((((((gastric diffused large B-cell lymphoma*[Title/Abstract]) OR (stomach diffused large B-cell lymphoma*[Title/Abstract])) OR (gastric DLBCL[Title/Abstract])) OR (stomach DLBCL[Title/Abstract])) OR (gastric diffused large B-cell lymphoma*[Text Word])) OR (stomach diffused large B-cell lymphoma*[Text Word])) OR (gastric DLBCL[Text Word])) OR (Stomach DLBCL[Text Word])) OR ((((gastric lymphoma[Title/Abstract]) OR (stomach lymphoma[Title/Abstract])) OR (gastric lymphoma[Text Word])) OR (stomach lymphoma[Text Word]))) OR ((((((gastric mucosa-associated lymphoid tissue lymphoma[Title/Abstract]) OR (stomach mucosa-associated lymphoid tissue lymphoma[Title/Abstract])) OR (gastric mucosa-associated lymphoid tissue lymphoma[Text Word])) OR (stomach mucosa-associated lymphoid tissue lymphoma[Text Word])) OR (MALT lymphoma[Text Word])) OR (Stomach MALT lymphoma[Text Word]))) OR (stomach neoplasms[MeSH Terms])))

Scopus

(TITLE-ABS-KEY("epstein barr virus associated gastric lymphoma ") OR ((TITLE-ABS-KEY("epstein barr virus") OR TITLE-ABS-KEY("ebv") OR TITLE-ABS-KEY("epv") OR TITLE-ABS-KEY("eb virus") OR TITLE-ABS-KEY("epstein virus") OR TITLE-ABS-KEY("human herpesvirus 4") OR TITLE-ABS-KEY("human herpes virus 4") OR TITLE-ABS-KEY("hhv 4") OR TITLE-ABS-KEY("infectious mononucleosis") OR TITLE-ABS-KEY("infectious mononucleosis virus")) AND ( TITLE-ABS-KEY("gastric tumour" ) OR TITLE-ABS-KEY("stomach tumour") OR TITLE-ABS-KEY("stomach lymphoma") OR TITLE-ABS-KEY("gastric lymphoma") OR TITLE-ABS-KEY("stomach neoplasia") OR TITLE-ABS-KEY("gastric neoplasia") OR TITLE-ABS-KEY("stomach neoplasm") OR TITLE-ABS-KEY("gastric neoplasm") OR TITLE-ABS-KEY("gastric DLBCL") OR TITLE-ABS-KEY("gastric MALT lymphoma") OR TITLE-ABS-KEY("stomach DLBCL") OR TITLE-ABS-KEY("gastric MALT lymphoma") OR TITLE-ABS-KEY("gastric diffused large B-cell lymphoma") OR TITLE-ABS-KEY("gastric mucosa-associated lymphoid tissue lymphoma") OR TITLE-ABS-KEY("stomach diffused large B-cell lymphoma") OR TITLE-ABS-KEY("gastric mucosa associated lymphoid tissue lymphoma")))

SciELO

((ab:(epstein barr associated gastric lymphoma)) OR (ti:(epstein barr associated gastric lymphoma)) OR (ab:(epstein barr associated stomach lymphoma)) OR (ti:(epstein barr associated stomach lymphoma)) OR (ti:(gastric lymphoma)) OR (ab:(gastric lymphoma)) OR (ti:(stomach lymphoma)) OR (ab:(stomach lymphoma)) OR (ti:(gastric neoplasm)) OR (ab:(gastric neoplasm)) OR (ti:(stomach neoplasm)) OR (ab:(stomach neoplasm)) OR (ti:(stomach dlbcl)) OR (ab:(stomach dlbcl)) OR (ti:(gastric dlbcl)) OR (ab:(gastric dlbcl)) OR (ti:(gastric malt lymphoma)) OR (ab:(gastric malt lymphoma)) AND (ti:(stomach malt lymphoma)) OR (ab:(stomach malt lymphoma)) OR (ti:(gastric diffused large b-cell lymphoma)) OR  (ab:(gastric diffused large b-cell lymphoma)) OR  (ti:(stomach diffused large b-cell lymphoma)) OR (ab:(stomach diffused large b-cell lymphoma)) OR (ti:(gastric mucosa associated lymphoid tissue lymphoma)) OR (ab:(gastric mucosa associated lymphoid tissue lymphoma)) OR (ti:(stomach mucosa associated lymphoid tissue lymphoma)) OR  (ab:(stomach mucosa associated lymphoid tissue lymphoma)) AND (ti:(epstein barr virus)) OR (ab:(epstein barr virus)) OR (ti:(eb virus)) OR (ab:(eb virus)) OR (ti:(epv)) OR (ab:(epv)) OR (ti:(ebv)) OR (ab:(ebv)) OR (ti:(hhv 4)) OR (ab:(hhv 4)) OR (ti:(human herpes virus 4)) OR (ab:(human herpes virus 4)) OR (ti:(human herpesvirus 4)) OR (ab:(human herpesvirus 4)) OR (ti:(infectious mononucleosis )) OR (ab:(infectious mononucleosis ))

**World of Science**

| **Search number** | **Search terms** |
| --- | --- |
| 26 | #25 OR #24 |
| 25 | TS=(epstein barr virus associated gastric lymphoma*) OR TI=(epstein barr virus associated gastric lymphoma*) |
| 24 | #23 AND #12 |
| 23 | #21 OR #20 OR #19 OR #18 OR #17 OR #16 OR #15 OR #14 OR #13 |
| 22 | TS=(infectious mononucleosis virus) OR TI=(infectious mononucleosis virus) |
| 21 | TS=(infectious mononucleosis) OR TI=(infectious mononucleosis) |
| 20 | TS=(epstein virus) OR TI=(epstein virus) |
| 19 | TS=(eb virus) OR TI=(eb virus) |
| 18 | TS=(epv) OR TI=(epv) |
| 17 | TS=(ebv) OR TI=(ebv) |
| 16 | TS=(hhv 4) OR TI=(hhv 4) |
| 15 | TS=(human herpes virus 4) OR TI=(human herpes virus 4) |
| 14 | TS=(human herpesvirus 4) OR TI=(human herpesvirus 4) |
| 13 | TS=(epstein barr virus) OR TI=(epstein barr virus) |
| 12 | #11 OR #10 OR #9 OR #8 OR #7 OR #6 OR #5 OR #4 OR #3 OR #2 OR #1 |
| 11 | TS=(gastric tumor*) OR TI=(gastric tumor*) OR TS=(gastric tumour*) OR TI=(gastric tumour*) |
| 10 | TS=(stomach tumor*) OR TI=(stomach tumor*) OR TS=(stomach tumour*) OR TI=(stomach tumour*) |
| 9 | TS=(gastric neoplasia*) OR TI=(gastric neoplasia*) |
| 8 | TS=(stomach neoplasia*) OR TI=(stomach neoplasia*) |
| 7 | TS=(gastric neoplasm*) OR TI=(gastric neoplasm*) |
| 6 | TS=(stomach neoplasm*) OR TI=(stomach neoplasm*) |
| 5 | TS=(stomach lymphoma*) OR TI=(stomach lymphoma*) |
| 4 | TS=(gastric lymphoma*) OR TI=(gastric lymphoma*) |
| 3 | TS=(stomach MALT lymphoma) OR TI=(stomach MALT lymphoma) OR TS=(stomach mucosa-associated lymphoid tissue lymphoma) OR TI=(stomach mucosa-associated lymphoid tissue lymphoma) OR TS=(gastric MALT lymphoma) OR TI=(gastric MALT lymphoma) OR TS=( gastric mucosa-associated lymphoid tissue lymphoma) OR TI=(gastric mucosa-associated lymphoid tissue lymphoma) OR TS=(stomach DLBCL) OR TI=(stomach DLBCL) OR TS=(gastric DLBCL) OR TI=(gastric DLBCL) OR TS=(gastric diffused large b-cell lymphoma) OR TI=(gastric diffused large b-cell lymphoma) OR TS=(stomach diffused large b-cell lymphoma) OR TI=(stomach diffused large b-cell lymphoma) |
| 2 | TS=(gastric lymphoma*) OR TI=(gastric lymphoma*) |
| 1 | TS=(stomach lymphoma*) OR TI=(stomach lymphoma*) |

Additional Figure 1: Inclusion and exclusion criteria flow chart

Records identified from PubMed, MEDLINE, Web of Science, SCOPUS, SciELO (Spanish)
(**n = 12,777**)

**Identification**

Duplicates removed

(**n = 5,423**)

**Screening**

Records screened after

removing duplicates
(**n = 7,354**)

**Records excluded based on title and abstract (n = 6,282)**

Not an original article (n = 1,400)

Cases less than 5 (n = 1,264)

Animal study (n = 92)

Laboratory work (n = 1,315)

Not EBV gastric lymphoma (n = 1,559)

Did not meet other criteria (n = 652)

**Eligibility**

Full-text articles

assessed for eligibility
(**n = 1,072**)

**Records excluded based on full article (n = 1,060)**

Not an original article (n = 90)

Cases less than 5 (n = 170)

Animal study (n = 8)

Laboratory work (n = 149)

Not EBV gastric lymphoma (n = 425)

No EBER or EBV prevalence detected using ISH (n = 7)

Did not meet other criteria (n = 211)

**Studies included (n = 12)**

**(MALT lymphoma = 10;**

**DLBCL = 11)**

**Inclusion**

**Abbreviations:** EBER ISH=EBV-encoded RNA in-situ hybridization; EBV=Epstein-Barr virus.
